# A Poly(ionic liquid) Gel Electrolyte for Efficient all Solid Electrochemical Double-Layer Capacitor

**DOI:** 10.1038/s41598-018-29028-y

**Published:** 2018-07-19

**Authors:** M. Taghavikish, S. Subianto, Y. Gu, X. Sun, X. S. Zhao, N. Roy Choudhury

**Affiliations:** 1 University of South Australia, Mawson Lakes Campus, Adelaide, South Australia Australia; 2 University of Queensland, Brisbane, Australia; 3 School of Engineering, RMIT University, Melbourne, Victoria 3001 Australia

## Abstract

Polyionic liquid based gels have stimulated significant interest due to their wide applications in flexible electronics, such as wearable electronics, roll-up displays, smart mobile devices and implantable biosensors. Novel supported liquid gel electrolyte using polymerisable ionic liquid and an acrylate monomer, has been developed in this work by entrapping ionic liquid during polymerisation instead of post polymerisation impregnation. The chemically crosslinked polyionic liquid gel electrolyte (PIL) is prepared using 2-hydroxyethylmethacrylate (HEMA) monomer and a polymerisable ionic liquid, 1,4-di(vinylimidazolium)butane bisbromide (DVIMBr) in an ionic liquid (IL- 1-butyl-3 methylimidazolium hexafluorophosphate) as the polymerisation solvent, which resulted in *in-situ* entrapment of the IL in the gel during polymerisation and crosslinking of the polymer. The supported liquid gel electrolyte (SLG) material was characterised with thermal analysis, infrared spectroscopy, and dynamic mechanical analysis, and was found to be stable with good mechanical properties. The electrochemical analysis showed that these chemically cross-linked PIL gel electrolyte-supported ILs are suitable for solid-state, flexible supercapacitor applications.

## Introduction

Electrochemical double-layer capacitors (EDLCs) store energy through reversible ion adsorption at high surface area electrode surfaces^1^. They have many advantageous properties such as high power density and long cycle life, making them a key energy storage system for many applications. However, the maximum energy of the EDLC is related to its capacitance and maximum operating voltage, therefore the challenge is to improve these parameters by delivering novel materials and configurations^2^. This can be done through increasing cell voltage, and as such much attention has been focused on electrolytes that would have sufficient thermal and electrochemical stability to operate at high voltage and over a range of temperatures.

Room temperature Ionic Liquids (IL) are organic salts that are liquid at room temperature without the presence of solvents^3^. They comprise solely of ions and are considered ‘green’ materials with some very interesting properties, as they are a good solvent for a wide variety of organic and inorganic materials, highly polar yet non-coordinating, non-volatile, and have tunable solubility and miscibility^4^. Currently, a variety of IL cations and anions combinations exists, but imidazolium cation based-ILs are of particular interest due to their high conductivity which has been attributed to the planarity of the cationic core of the imidazolium ring. The anions, on the other hand, generally determine properties such as miscibility, with fluorinated anions resulting in ILs that are immiscible with water.

There are many advantages to the use of ILs as EDLC electrolytes. They have very wide voltage windows^5^ (up to 6 V) and large intrinsic capacitance, making them good materials for high energy electrochemical devices^2,3^. Their incombustibility and low vapour pressure also make them less sensitive to elevated temperatures. However, ILs are still liquids. The use of a liquid electrolyte leads to a number of practical and environmental drawbacks, such as the rigid metal casing required for containment adding weight and restricting possible device geometries, and the possibility of leakage [Lu *et al*., 2014]. By immobilising ILs in solid media such as polymers and silica gels, solid-state ILs, namely ionogels, can be obtained. However, gel polymer electrolytes based on ionic liquids and linear polymers usually exhibit poor mechanical properties, including both strengths and flexibility, because of their few polymer chain entanglements separated by small molecules. Also supported liquid membrane prepared by post polymerization impregnation of IL is an alternative approach for EDLC. However, it often poses challenge in the diffusion related immobilization process. To achieve supported IL based flexible polymer electrolytes, crosslinking strategies have been proposed in recent years. However, current materials are generally inert or doped with salts of low viscosity ionic liquids, and the choice of polymers is often restricted by the miscibility of certain polymers in ILs especially at high degrees of polymerisation. Another possibility is the use of porous matrices, however such materials risk long-term stability due to leaching of the IL over time. The *in-situ* encapsulation of IL involves simultaneous formation of a three-dimensional (3D) network and entrapment, which percolates throughout the matrix and is responsible for the solid-like behaviour of the ionogel. The polymer network thus acts like a sponge, with the holes filled with the IL. Because the holes are much larger than the ions, the ion mobility is comparable to that in the pure IL state. Thus in this work, we explored the potential of novel supported liquid gel electrolyte. In this regard, poly ionic liquid is an attractive alternative to traditional polymer materials. PILs have good thermal and electrochemical stability, and they can also be expected to have good miscibility and compatibility with ILs due to their common structure^6^. These properties make them attractive for applications such as gel matrices as they can be loaded with conducting materials such as ILs^7^. The gel scaffold serves both to constrain the IL in a solid form, as well as to provide a physical barrier between the electrodes, preventing short circuits between the two electrodes. Thus on one hand, this entrapment eliminates the risk of any leakage, on the other hand, ILs have ability to plasticize the polymer matrixes or network and increase the mobility of ions in electrolytes. As a result, there is no need to put a separator between the two electrodes in fabricating EDLC devices with ionogel electrolyte. This has potential to lower the cost of the EDLC cells.

Despite these potential advantages such as higher conductivity of PILs compared to non-ionic polymers such as PVA^8^, thus far the use of poly(ionic liquid) materials for EDLC electrolytes have not been studied. In this paper we report for the first time the fabrication of a PIL (1,4-di(vinylimidazolium)butane bisbromide DVIMBr) and 2-hydroxyethylmethacrylate (HEMA) based electrolyte in ionic liquid solvent. The surge of interest in using ionic liquids (ILs) as reaction media for organic reactions has so far led to many advantages including not only ability to replace volatile organic solvents, but also to incorporate advantageous properties of the ionic liquids themselves. The PIL serves as a crosslinker to HEMA and would enhance the ionic matrix, with the ionic imidazolium group of the PIL complementing the hydroxyl groups of HEMA. This composition would offer a good percolation of polar groups and salt solvation of the free ionic liquid, which should possess low viscosity and high mobility in order to achieve the optimum performance^1^. These PIL gels are expected to show promising results as new electrolyte materials for supercapacitors. The gel scaffold can serve both to constrain the liquid in a solid form, as well as to provide a physical barrier between the electrodes, preventing short circuits. Thus, there is no need to put a separator between the two electrodes in fabricating SC devices with ion gels as the electrolytes. Because of these desirable properties, ion gel energy storage devices are safer, lighter, theoretically more cost-effective (no separator needed), and more amenable to future flexible applications.

## Results and Discussion

### *S*ynthesis and characterisation of the PIL gel electrolyte: Crosslinking HEMA with DVIMBr with *in-situ* entrapment of IL

**Figure 1:**
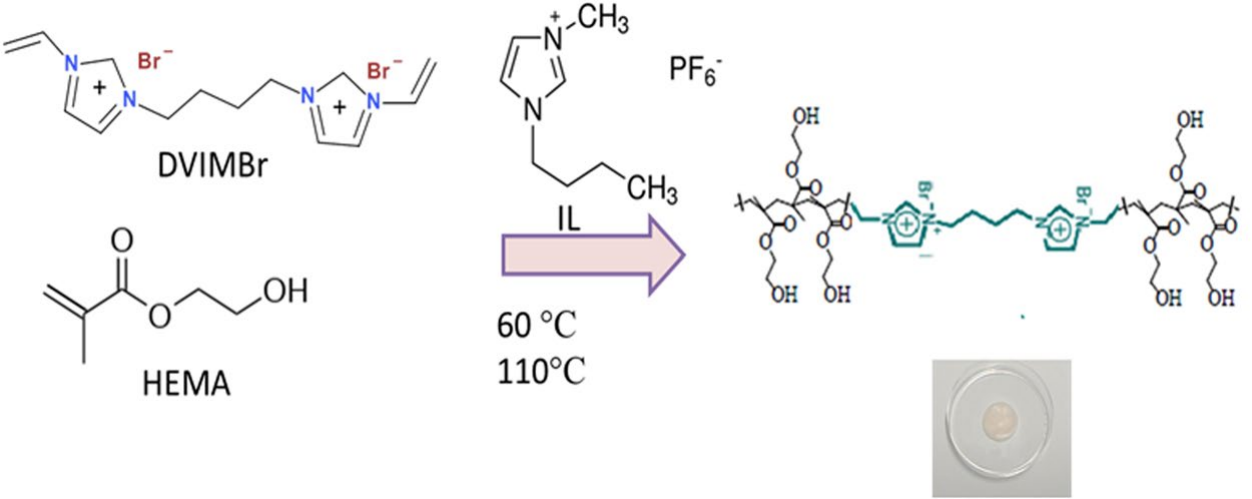
Schematics of Polymerisation of PIL Gel Electrolyte.

The PIL used in this study is a divinyl monomer, 1,4-di(vinylimidazolium)butane bisbromide (DVIMBr) (Fig. 1), based on the imidazolium cation for 2-hydroxyethylmethacrylate (HEMA). Polymerisation of HEMA in presence of DVIMBr (where the DVIMBr acts as a crosslinker to HEMA) was successfully done using 1,1-azobis(cyanovaleric acid) (ACVA) as the thermal initiator, with ACVA being chosen due to its excellent miscibility with HEMA and the PIL. A small amount of MeOH was used to aid the solubilisation of DVIMBr, as it did not readily dissolve in HEMA. Thermal polymerisation and crosslinking of HEMA-DVIMBr resulted in a clear, glassy material, which was rigid due to the crosslinking through DVIMBr and hydrogen bonding of HEMA. Unlike ILs, PILs are solids (albeit often with low glass transition temperatures) and are similar to ordinary polymers^9^. This means that due to the polymeric nature of the cation (in DVIMBr), the PIL gel can behave essentially as a single ion conductor and its conductivity depends largely on the mobility of the anion alone^10^. Thus, the electrolyte gel is formed using ionic liquid (1-butyl-3methylimidazolium hexafluorophosphate) as a solvent. As the polymerisation progresses, the free ionic liquid is immobilised inside the gel and the HEMA-DVIMBr forms a crosslinked network, thus forming the final gel containing the ionic liquid. This was possible as ionic liquid acts as a good solvent for both the precursors, and has been shown to have a beneficial effect on free radical polymerisation^11^.

**Figure 2:**
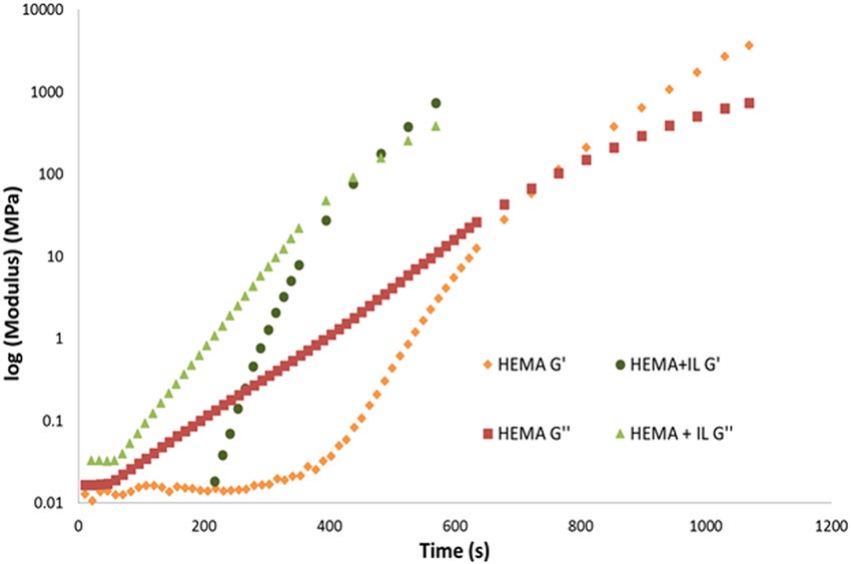
Logarithmic plot of the elastic modulus (G′) and viscous modulus (G′′) of HEMA and HEMA-ionic liquid during polymerisation.

The polymerisation of HEMA and crosslinking with DVIMBr was studied by rheology, where polymerisation results in an increase in the elastic modulus (G′) and the viscous modulus (G′′). The crossover point between these two values indicates the gel point where the material shows a more elastic rather than a viscous characteristics. Figure 2 shows the viscous modulus and the elastic modulus of HEMA polymerisation at 60 °C with and without IL. Interestingly, the addition of IL increases the rate of polymerisation of HEMA (as shown by a shorter time taken to reach the gel point), with the IL-containing sample showing reaching the gel point almost twice as rapidly as the sample without IL. This shows that IL is a good solvent for polymerisation of HEMA, and that its use increased the rate of polymerisation. This has also been previously reported in the literature for various polymer systems including methacrylates^12^.

The values of the viscous and elastic moduli at the gel points remain quite similar between the two samples, although slightly lower for HEMA with IL at 91 MPa compared to 102 MPa for HEMA alone. This 10% reduction in modulus at the gel point indicates that the IL has only a weak plasticizing effect on HEMA, which may indicate a lack of interaction between HEMA and the IL. This lower modulus value of the sample with IL is due to reduction in crosslink density of the sample in presence of IL and lack of strong interaction between the hydroxyl groups of HEMA and the imidazolium group of ionic liquid.

**Figure 3:**
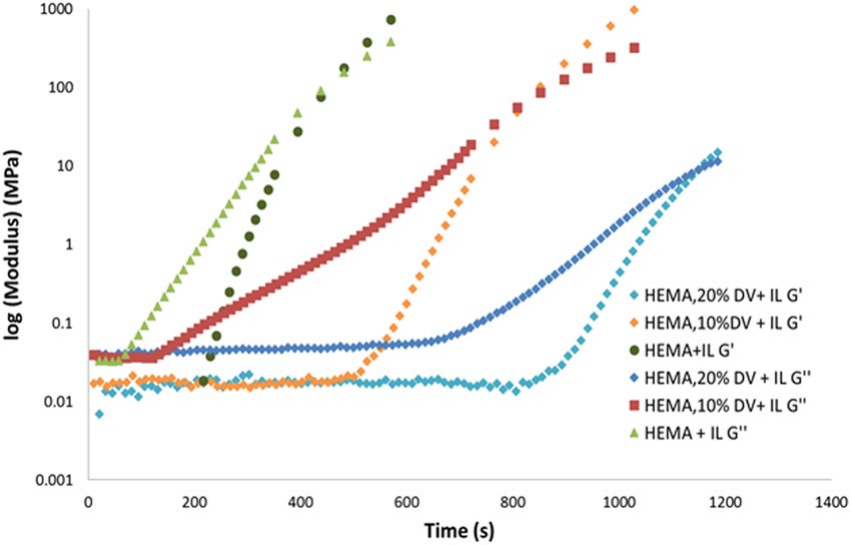
Logarithmic plot of the elastic modulus (G′) and viscous modulus (G′′)of the HEMA-DVIMBr copolymer in the presence of excess Ionic Liquid (75 wt%).

When DVIMBr is present, and in the presence of the same quantity of excess IL (75 wt%), the rate of polymerisation appears dependent on DVIMBr concentration. As can be seen in Fig. 3, the polymerisation behaviour depends on the proportion of DVIMBr in the batch, with higher DVIMBr concentration resulting in slower kinetics. Since the two vinyl groups of DVIMBr enables crosslinking of the polymer, it is likely that increasing the DVIMBr content resulted in a slower polymerisation time due to a more crosslinked network structure formation that slows down diffusion of unreacted monomers, thus slowing down the polymerisation process. The crosslinked network creates greater diffusion limitation for the free radicals to terminate. The maximum reaction rate initially increases for HEMA which decreases as the DVIMBR content increases. It indicates that the overall reaction although enhanced after an induction period by crosslinking aided gel effect and then retarded due to limited mobility of unreacted monomers. Similar observation has been made for HEMA polymersation and crosslinking with ethylene glycol diacrylate crosslinker^13^. This is further exacerbated by the viscosity of the IL reaction medium. Interestingly, the modulus at the gel point is also lower for higher DVIMBr content samples despite the crosslinking, with the gel point occurring at 91 MPa for HEMA + IL, and when DVIMBr is present the gel point occurs at 56 and 9 MPa for 10% and 20% DVIMBr content respectively. This may be attributed to a couple of factors: Firstly, due to the greater degree of crosslinking the gel point may arrive at a lower degree of polymerisation compared to less crosslinked samples, and thus the gel point itself may be encountered at a lower modulus despite the final material being more crosslinked. Secondly, larger content of DVIMBr may allow greater amount of ionic liquids to be incorporated within the polymer structure, thus resulting in a softer, more plasticized material at the gel point.

**Figure 4:**
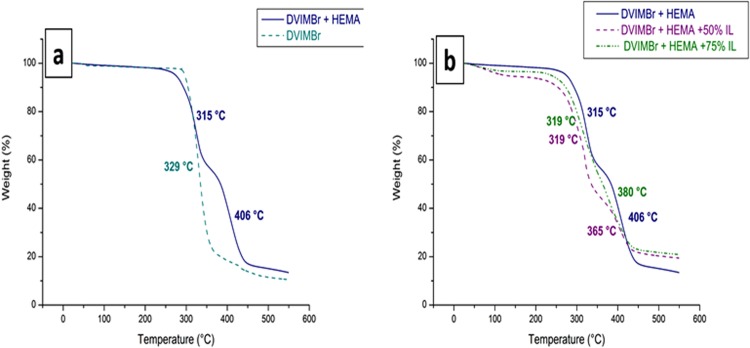
TGA thermogram of the (**a**) HEMA-DVIMBr copolymer (without IL) and homopolymerised DVIMBr, (**b**) HEMA-DVIMBr-IL gel containing, 0, 50, and 75 wt% IL.

Figure 4a shows thermogravimetric analysis of the DVIMBr crosslinked HEMA polymer (polymerization without free IL solvent) which shows two degradation temperatures for the polymer, the first one at 315 °C due to DVIMBr and the second one at 406 °C corresponding to crosslinked HEMA. The thermal degradation of polyHEMA has been reported in the literature to occur in two stages, with the first mass loss at 160–200 °C and a second one at above 300 °C^14,15^. The crosslinked HEMA did not show any degradation below 300 °C, which indicates an increased thermal stability of the HEMA-DVIMBr polymer due to the crosslinking by the thermally stable DVIMBr, as poly(HEMA) degradation below 300 °C have been attributed to depolymerisation^14^. The higher thermal stability in crosslinked networks results from change in thermal conductivity and specific heat capacity of the materials. The second degradation peak at 406 °C also indicates strong interaction between the imidazolium groups of DVIMBr and the hydroxyl groups of HEMA, as it has been previously observed in ionomers that the interaction between acidic groups and IL significantly increases their thermal stability^16^, with a similar effect to converting those acidic groups to their corresponding salt form. The TGA thermogram also shows that under the conditions used, the polymerisation has completed as there was only the degradation temperature of the HEMA polymer observed, and no trapped, unreacted HEMA (boiling point of 205 °C) was detected.

### Effect of IL content on the crosslinked HEMA

Following this, a study was done to determine the effect of ionic liquid concentration in polymerized and crosslinked HEMA. However, no gelation study was undertaken using rheology at various IL ratios, as it did not show much difference with the lower content (50%). Therefore, all property studies have been carried out with highest IL content mainly. However, to investigate the effect of IL, the proportion of DVIMBr to HEMA was kept constant at 10:90 (w/w), and the monomer:initiator ratio used was kept at a 0.05 to achieve a high molecular weight material while the IL content was varied up to 75 wt%. The incorporation of the IL can be confirmed through TGA analysis in Fig. 4b which shows an increase in the residue at high temperature (above 450 °C) due to the IL. The IL has a peak decomposition temperature at 415 °C (SI Fig. 3). The sample with 75% IL shows a slightly higher amount of residue compared to the sample with 50% IL, however the overall similar levels of residue amount (Tables 1, 19.5 and 20.5%) for both 50 and 75% IL indicates saturation of the gel (maximum IL loading) has been reached, and this is likely determined by the crosslinking in the material. The higher amount of residue is attributed to the nitrogen and phosphorous contents of the IL and PIL. The addition of IL also reduces the second degradation peak compared to the sample without IL, indicating that the second degradation is due to a strong interaction between HEMA-DVIMBr and IL where the hydroxyl group of HEMA may interact with the PIL, and thus the addition of IL solvates the DVIMBr, reducing this interaction and thus at high IL content the second degradation is not as prominent. The high residue content is attributed to both nitrogen and phosphorous contents of the IL and the PIL in the sample.

**Table 1:** TGA results of crosslinked poly HEMA with IL content.

Sample	Comp 1 Decomp. Temp. °C	Comp 2 Decomp. Temp. °C	Residue %
DVIMBr	329	—	10
HEMA-DVIMBr	315	406	12
HEMA-DVIMBr-50%IL	319	365	19.5
HEMA-DVIMBr-70%IL	319	380	20.5

When incorporated in the HEMA-DVIMBr crosslinked polymer, the ionic liquid serves as a plasticizer and it can be seen that although the HEMA:DVIMBr copolymer was glassy due to the crosslinking by DVIMBr, the IL-containing gels are elastic, and visual differences can be observed in the resultant material. The sample without IL was clear, while the IL-containing samples were cloudy (Fig. 1). Preliminary electrochemical testing (which will be discussed later) shows that the capacitance and conductivity of the gels with increasing IL content, with the samples containing no IL showing poor conductivity. However, the sample with the highest IL content under compression shows marginal loss of unbound IL from the sample. TGA analysis of this sample shows only one broad degradation peak, however, indicating a strong interaction between the IL and the DVIMBr in the matrix. This indicates that the leaching is due to oversaturation, and not a lack of interaction between the IL and the HEMA-DVIMBr polymer network.

### Effect of DVIMBr content on the gel electrolyte

This study was conducted through increasing the DVIMBr:HEMA ratio, as it is expected that materials with greater DVIMBr content would be able to hold an increased amount of IL. To that end, samples were made containing 10, 20, and 30% DVIMBr and 75 w/w% IL in all cases. Figure 3 shows the representative plot of elastic and viscous modulus for poly HEMA at different DVIMBr corsslinker ratio. From the plot is is evident that the occurrence of the major gel formation shifts in the order HEMA < DVB 10% < DVB 20% towards the increasing initial concentrations of the crosslinking agent in the monomer mixture. When the crosslinking agent is present, initial gel formation is delayed with higher amount taking longer set in time and rises markedly slowly at higher quantities of the crosslinking agent. However, after polymerization, the gels show uniform surface. The gels containing 30% DVIMBr did not show significant leaching of IL after polymerization, indicating that the extra DVIMBr improves IL retention and allows extra IL to be incorporated into the gel. Leaching experiments with the samples kept under ambient conditions in a dessicator show some loss of free or unbound IL over time, however, the mass loss between the different samples were very low (1–3%) and were within the error margin of each other, which shows the low amount of leaching under ambient conditions. It is likely that the leaching polymerization of these gels would be affected differently when used as an electrolyte in a supercapacitor (due to the charge-discharge cycle) however without external stressors the gels appear stable with minimal leaching.

**Figure 5:**
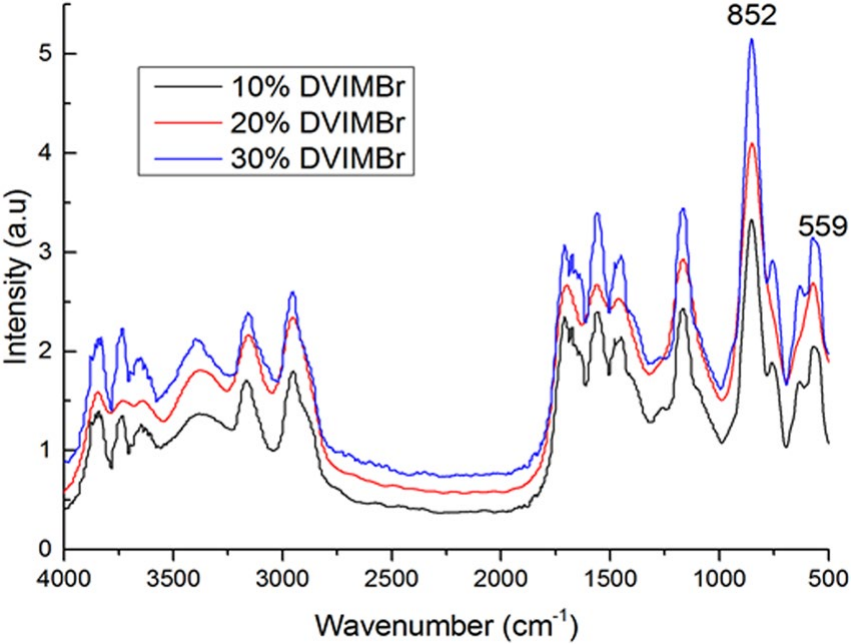
FTIR Spectra of HEMA-DVIMBR-IL gels with increasing DVIMBr content and 75% IL.

Furthermore, PA-FTIR analyses of the samples in Fig. 5 show that the peaks at 852 and 559 cm^−1^ due to the PF_6_^−^ counterion in the IL were quite similar for all three samples, only increasing very slightly with greater DVIMBr content. Elemental analyses of the samples show only a slight increase in the nitrogen content of the sample from 6.8% to 8.5% between the samples containing 10 and 30% DVIMBr, which is consistent with increased DVIMBr loading as the nitrogen content of the sample would be higher due to the imidazolium moiety found both in DVIMBr and the free IL. This slight increase indicates that the baseline loadings of IL between the samples are similar after synthesis amongst the three samples. Regardless, higher DVIMBr content would mean higher degree of crosslinking, and such samples are expected to be more mechanically stable compared to sample with less crosslinking.

**Figure 6:**
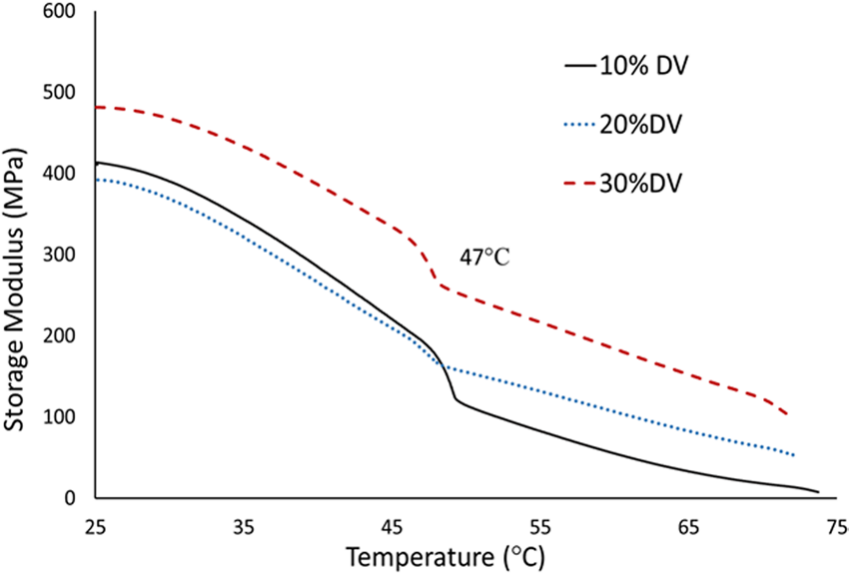
Dynamic mechanical analysis of gels containing 10, 20 and 30% DVIMBr and 75 wt% IL.

To investigate the mechanical properties of these gels, dynamic mechanical analysis (DMA) was performed. The results in Fig. 6 show that increasing the DVIMBr content increases the mechanical strength of the gel at high temperature (70 °C) due to increasing the number of crosslinks. The crosslink density (n) of the samples, which quantifies the number of moles of active network chains per unit volume (mol/m^3^), was calculated for the samples according to the rubber elasticity theory using the expression: n = E′/3RT = ρ/Mc, or E = 3ρRT/Mc where Mc represents the molecular weight between crosslinks (g/mol), E′ is the tensile storage modulus (Mpa) as measured from DMA experiments, R is the universal gas constant (8.314 J/mol.K), T is the absolute temperature (298 K) and ρ is the density of the samples. As the 30% DVIMBR sample exhibited a higher storage modulus value than that of 10 and 20% samples, consequently the 30% DVIMBR sample had a higher crosslink density. Table 2 reports the Mc values and modulus data from DMA. Using the density 1.15, the Mc values for the samples were calculated and they are in the range of 17.8 g/mol for 30% DVIMBR sample; 21.3 g/mol for 20% DVIMBR sample, to 22.5 g/mol for 10% DVIMBR sample respectively. As the Mc is inversely proportional to the E′, 30% DVIMBR sample has a lower molecular weight between crosslinks when compared to the 10 or 20% samples, which accounts for the higher modulus values for the 30% sample. Under ambient conditions, all three samples show increasing trend in modulus, with the 30% DVIMBr sample showing higher modulus than the 10 or 20% sample with an increase from 400 to 480 Mpa. This increase was more evident at 70 °C where the modulus were 14, 54, and 104 MPa respectively for samples containing 10, 20, and 30% DVIMBr. This indicates that the increased crosslinking with increased DVIMBr content has a significant effect on mechanical integrity at higher temperature, and thus by extension samples with higher DVIMBr content may be more suitable for applications that require such environment despite the similar IL loading in all three samples. The crosslinked samples also show a transition at around 47 °C which is likely due to DVIMBr rather than HEMA as such transition have not been reported in the thermal analysis of HEMA^14,15^. Rather than a glass transition, this may be a cluster transition due to the presence of ionic clusters of DVIMBr. Such transition can be observed in certain ionomers where at higher temperatures there is a loss of long range ordering in the ionic domains^17^.

**Table 2:** Effect of crosslinking on the mechanical properties and the Tg of the PIL gels.

Sample	Mc g/mol	E′ @25 °C MPa	E′@70 °C MPa	Tg in °C
DVIMBr 10%	22.5	400	14	49
DVIMBr -20%	21.3	420	54	48
DVIMBr -30%	17.8	480	104	47

### Electrochemical Analysis

Electrochemical analyses were performed to investigate the suitability of these PIL gels as EDLC electrolytes. Samples were prepared with a fixed 10% DVIMBr loading and IL loadings of 0, 23, 50, and 75% in order to study the effect of IL content. Cyclic Voltammetry results of the samples showed that in the absence of extra ionic liquid, the conductivity in the sample was poor. This is attributed to the relatively low PIL content in the gel (10%), which means that there is insufficient PIL moiety to achieve charge percolation, and in addition the ionic groups in the PIL are crosslinked and thus immobilised in the polymer matrix, unable to transfer charges. Samples with 0 and 23% IL content show very small capacitance values, which are attributed to a lack of conductivity. When samples with 23, 50, and 75 wt% of IL were analysed by electrochemical impedance measurements, they showed increased conductivity and capacitance with increasing IL content, with the highest capacitance and conductivity observed for the sample with the highest IL content. Recently, Pandey *et al*.^18^ also investigated symmetric electrical double layer capacitors (EDLCs) with ionic liquid based magnesium ion conducting gel polymer electrolytes and activated charcoal (AC) electrodes. The authors noted that the incorporation of magnesium salt and EC-PC co-solvent in gel polymer electrolytes play a significant role in the dramatic improvement of the EDLCs’ characteristics. However, they used activated charcoal as electrode material.

**Figure 7:**
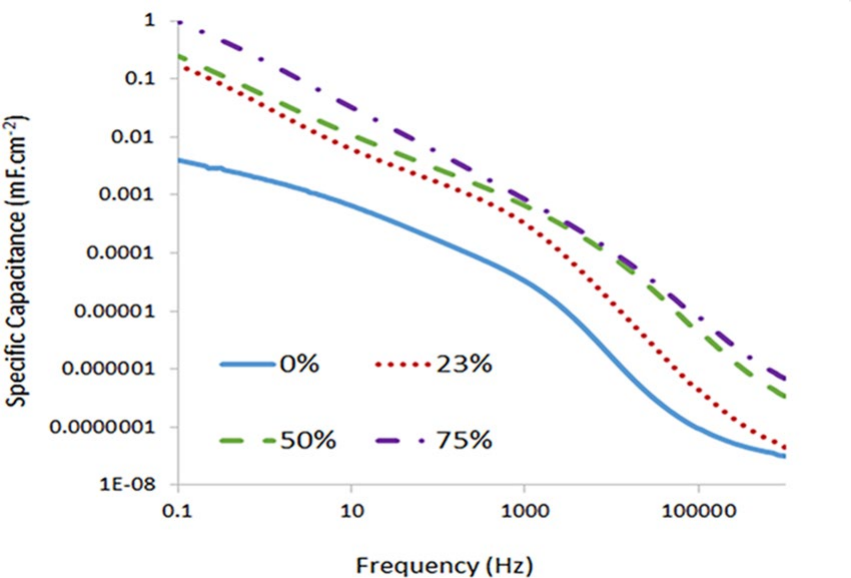
Specific capacitance of the samples containing 10% DVIMBr calculated through impedance.

Figure 7 shows the specific capacitance vs frequency calculated from the impedance using the relationship

1 $${{C}}_{{specific}}=\frac{4}{2{\pi }\mathrm{Af}(-{Z}^{\prime\prime} )}$$

where Cspecific (in F/m^2^) changes with frequency, f (in Hz). A (in m^2^) is the total surface area of both electrodes, and Z′′ (in ohm) is the imaginary part of impedance. By multiplying a factor of 4, the capacitance of a symmetric device could be converted into the specific capacitance on a single electrode. As can be seen, the samples exhibit potential for capacitor at frequency below 10 Hz, and the phase evolutions of the samples (See Supporting Information) also show low phase angle indicative of a resistive rather than capacitive nature of the samples. This is attributed to the materials conductivity being diffusion controlled and dependent on electrolyte permeation rate, as the conduction mechanism will most likely depends on the diffusion of free IL through the material.

**Figure 8:**
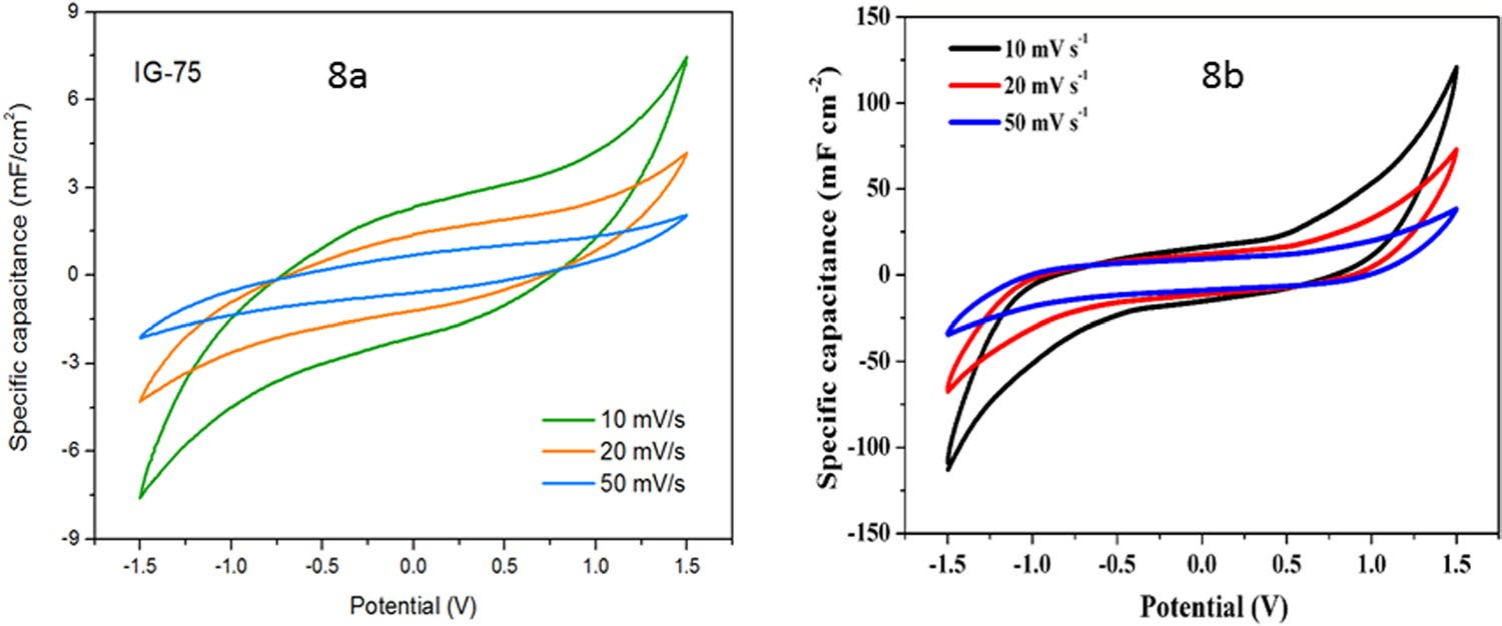
(**a**) Cyclic Voltammetry of the sample containing 10% DVIMBr and 75% IL; (**b**) CV curves of a supercapacitor cell fabricated with a gel containing 10% DVIMBr and 75% IL as electrolyte and commercial CNTs as both electrodes.

Cyclic voltammetry measurements show that samples with higher IL content have higher capacitance, with the sample without free IL showing very little capacitance due to a lack of conductivity. The sample with 75% IL in Fig. 8 is most promising due to its best conductivity, and shows a large potential window of around 3 V. As the gel was made *ex-situ* and then assembled into a supercapacitor for measurement, there was no skin effect^19^ (where a layer of IL coats the electrode regardless of electrolyte composition) observed and the IL content significantly affects the capacitance of the device.

**Figure 9:**
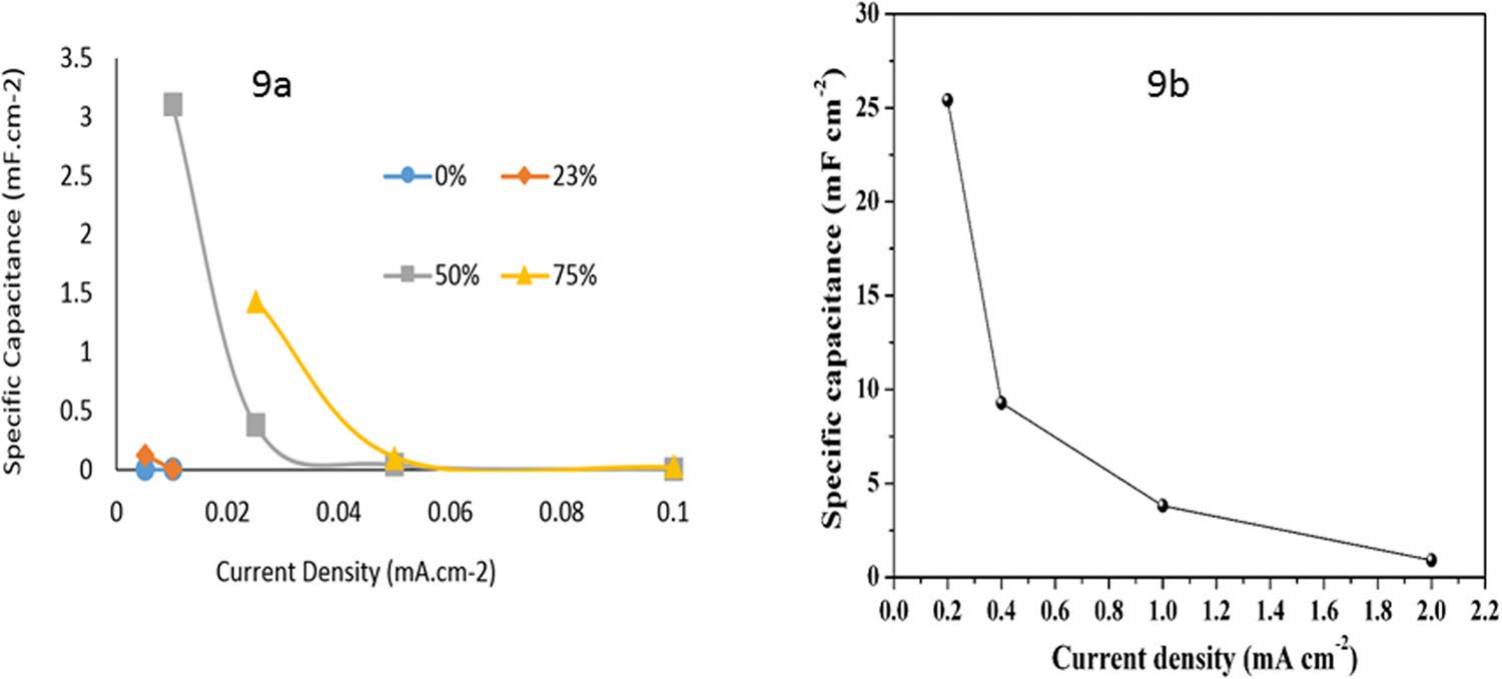
(**a)** Effect of IL content on the specific capacitance of the samples with current density (obtained from GCD curves). (**b**) The specific capacitance as a function of current density for a supercapacitor cell fabricated with a gel containing 10% DVIMBr and 75% IL as electrolyte and commercial CNTs as both electrodes.

The samples were then subjected to Galvanostatic Charge-Discharge (GCD) at current densities between 0.005 and 0.1 mA.cm^−2^ (see Supporting Information). Figure 9 shows the capacitance vs current density of the samples, which shows some differences with the capacitance values obtained through impedance analysis. Firstly, there is a significant increase between 23 and 50%, where the capacitance increased by an order of magnitude whereas the values are more similar in impedance analysis. This may indicate the percolation threshold for the charges, since the GCD analysis requires current flow in the material, and thus conductivity of the material would significantly influence the result. As can be seen in Fig. 9a, the capacitance values are very low for both 0 and 23% IL loading, which indicates that at 23% IL loading there was insufficient IL to ensure connectivity of the IL-rich domains within the material, and thus a lack of conductivity and low capacitance close to the sample without IL. The large increase between 23 and 50% IL indicates that at 50% IL the percolation threshold has been reached and there was sufficient connectivity between the IL domains for charge transport, thus resulting in higher capacitance values obtained through GCD. The sample with 75% IL showed the highest capacitance, but at high current densities this value decreased to that similar to the sample with 50% IL. We have also tested the electrocapacitive property of the gel electrolyte using commercially available carbon nanotubes (CNTs). The results are shown in Fig. 8b. The specific capacitance of the sample containing 10% DVIMBr and 75% IL using commercial CNTs as electrode materials versus current density obtained from the GCD method is shown in Fig. 9a. The specific capacitance as a function of current density for a supercapacitor cell fabricated with a gel containing 10% DVIMBr and 75% IL as electrolyte and commercial CNTs as both electrodes is shown in Fig. 9b. The capacitances reached 25.4 mF cm^−2^ at 0.2 mA cm^−2^ and 3.81 mF cm^−2^ at 1 mA cm^−2^, respectively, which are indeed higher than that when nonporous graphite was used as the electrodes. The GCD curves at various current densities between 0.2~2 mA cm-2 are shown in the Supporting Information (Fig. S6).

**Figure 10:**
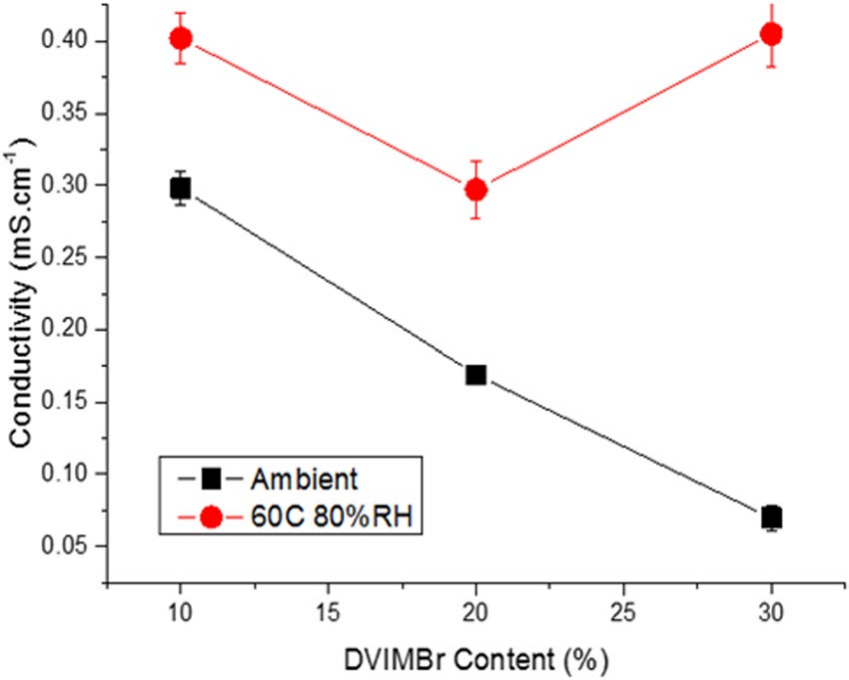
Conductivity of gels containing 10, 20, and 30% DVIMBr.

To further investigate this effect, samples were made with increased DVIMBr content to investigate the effect of DVIMBr loading on gel conductivity. Bulk samples with 10, 20, and 30% DVIMBr and a fixed 75 wt% IL solvent were analysed by electrochemical impedance spectroscopy in order to measure their conductivity under both ambient conditions and at elevated temperature. As can be seen in Fig. 10, the gels show conductivity values of around 0.3 to 0.07 mS.cm-1 under ambient conditions, with a decreasing trend with increasing DVIMBr content. This value is certainly lower than the acrylate gel based supercapacitor device prepared by Qin *et al*.^20^, where the electrolyte showed combination of robust elastic modulus (210 kPa) and reasonably high ionic conductivity (3.2 mS/cm). However, Pazner *et al*.^19^ also studied Poly(Ethylene Glycol) Diacrylate-Supported Ionogels where the authors noted that the ionic conductivity of the 44.7 wt % ionogel (0.47 mS/cm) is approximately a factor of 20 times lower than that of the neat ionic liquid. In the present case, modulus values are relatively higher (double) at ambient temperature indicating the rigid nature of the gel electrolyte and related charge transport issue. According to the authors, although the elastic modulus of those solid electrolyte materials varied by more than 4 orders of magnitude within the composition range they studied, concomitant changes in gel ionic conductivity and double layer capacitance were much less dramatic. However in our case, the conductivity value is higher than that observed by them. It can be clearly seen that the present ionogel electrolyte performed excellently in terms of capacitance, with some 75 wt.% loading of the IL, the ionogel EDLC exhibited a capacitance of 55 μF/cm2, which is five-time higher than that reported by Pazner *et al*.^19^. It must be pointed out that the electrodes used in our major study were non-porous graphite. If highly porous carbon electrode are used, it is expected that the capacitance can be significantly enhanced. While ionic conductivity is dependent upon ion concentration, however, the relationship between ionic conductivity and network structure depends on both the concentration of ionic group, mobility of charge carriers, and network flexibility. This indicates that the conductivity of the gels were more affected by IL transport^21^ and DVIMBr content, which is attributed to the ionic moieties in the DVIMBr being fixed and thus are not as effective in transferring charges. The lower conductivity for higher DVIMBr content is attributed to the more rigid structure of gels with higher DVIMBr content, where higher number of crosslinks may reduce diffusion of IL through the gel. When the temperature was increased to 60 °C (at 80% RH), the gels show an increase in conductivity to similar values for the three gels (0.4 ms/cm for 30% DVIMBr). This is attributed to the gel structures becoming softer at higher temperature as seen in previous DMA results, where the storage modulus of the gels decreased with increasing temperature. This would allow greater mobility of IL through the gel, resulting in the higher conductivity observed. Under this condition, the gel conductivity would more likely depend on IL loading and thus the gels show conductivity values that are closer to each other. The increase was more pronounced for the sample with 30% DVIMBr as it shows the lowest conductivity under ambient condition, however, at elevated temperature it shows the same conductivity as the sample with 10% DVIMBr. Currently, more work is underway to create long chain polymerisable ionic liquid crosslinkers to increase the flexibility of the gel electrolyte and achieve higher performance and will be reported in our future communication.

## Conclusions

Using a cross-linked polymer matrix as support for IL, freestanding PIL gel materials were synthesized by *in-situ* entrapment of IL during polymerization and crosslinking. The polymerization and crosslinking of HEMA with the polymerisable ionic liquid was seen to be facilitated by the IL solvent, which also results in the IL becoming directly entrapped upon crosslinked network formation. FTIR analysis shows the presence of free IL within the gel, and TGA shows good thermal stability of these gels upon IL incorporation. The gels show good mechanical properties and stability under ambient conditions and up to 70 °C. Electrochemical tests of these gel materials as supercapacitor electrolytes showed promising results, with a specific capacitance of 1.5–3 mF.cm^−2^ from galvanostatic charge-discharge curves. Given the mechanical properties of these gels are highly suitable for the EDLC, future work is underway to enhance the flexibility and the charge transport mobility to match the EDLC characteristics.

## Methods

1,4-dibromobutane, 1-vinylimidazole, 2-hydroxyethylmethacrylate (HEMA) and 1,1-azobis(cyanovaleric acid) (ACVA) were purchased from Aldrich (Australia) and used as received. The ionic liquid, 1-butyl-3-methylimidazolium hexafluorophosphate was purchased from TCI chemicals (Japan) and used as received. The PIL monomer, 1,4-di(vinylimidazolium)butane bisbromide (DVIMBr) was polymerized based on procedures reported elsewhere^7^. In a typical synthesis, a mixture containing of 2:1 molar ratio of 1-vinylimidazole to 1,4-dibromobutane was stirred in methanol at 60 °C for 15 h. To achieve a homogeneous mixture, the two ionic liquids are mixed with each other which was then added to HEMA and ACVA (initiator) for polymerization and crosslinking. After cooling down, the reaction mixture was added dropwise into 1 L of diethyl ether. The white precipitate was then filtered off and dried at room temperature to a constant weight (Yield: 68%). The NMR and IR data of the synthesized material is given in the supporting information (ESI 1, 2). 1H NMR (300 MHz, D2O, δ, ppm): 8.98 (2H), 7.69 (2H), 7.49 (2H), 7.04 (2H), 5.75 (2H), 5.33 (2H), 4.21 (4H), 1.87 (4H). (ESI-1).

The PIL gel was fabricated through thermal polymerization of the monomers in the presence of ionic liquid. DVIMBr was dissolved in minimum quantity of MeOH, and HEMA, ACVA, and IL was then added to the solution. The solution was then cast onto Teflon mould to fabricate discs of 20 mm diameter and 1, 0.5, and 0.3 mm thicknesses and put under vacuum at room temperature for 10 minutes to remove the methanol. The solution was then thermally polymerized first at 60 °C for 30 minutes and then at 110 °C for 30 minutes, after which the gel was removed from the mould and excess IL was wiped off with a laboratory wipe. Elemental analysis of the sample was performed by the University of Queensland Microanalytical Services.

Thermogravimetric analysis (TGA-model 2950) was performed using a TA Instruments Discovery TGA with an aluminium pan. Samples were subjected to a 10 °C/min heating rate from 100 °C to 550 °C under a nitrogen atmosphere. Infrared spectra were acquired using Nicolet Magna-IR Spectrometer 750 in photo-acoustic mode, with 256 numbers of scans and using a carbon black reference. The polymerization process was investigated using a Rheolyst 1000 N controlled stress rheometer (TA Instruments Delaware, U.S.A.). The measurements were undertaken at 60 °C using a 40 mm cone and plate geometry. The sample was placed between the fixed Peltier plate and a rotating cone attached to the driving motor spindle, and the measurements were performed under oscillation mode with a constant frequency of 1 Hz and 2.5% strain. Dynamic Mechanical Analysis (DMA) was performed using a TA Instruments Q-800 DMA with a constant humidity accessory. The samples were measured as rectangular films of approximately 20 × 5 × 1 mm dimension in a two-point clamp under a frequency sweep method with a static force of 0.01 N and a frequency of 10 Hz.

The PIL gel discs were used for electrochemical measurements in a two-electrode (symmetric) cell device. The discs were sandwiched by two graphite films (2.0 cm diameter × 0.05 cm thickness). Stainless steel meshes were applied as the current collectors. Electrochemical properties were measured on an Autolab PGSTAT302N electrochemical workstation at room tempserature under ambient conditions. Cyclic voltammetry (CV) and galvanostatic charge-discharge (GCD) tests were measured at different scan rates and current densities, respectively. Electrochemical impedance spectroscopy (EIS) was recorded over the frequency range 0.1 Hz to 1 MHz with a sinusoidal voltage amplitude of 10 mV superimposed on 0 V DC (vs. open circuit potential). Conductivity measurements on bulk gel samples were performed by in-plane electrochemical impedance. The sample was cut into strips and clamped in Teflon clamp with platinum electrodes spaced 1 cm apart. The resistance values were obtained from the impedance measurement and was used to calculate conductivity using the relationship δ = L/RA where L is the spacing between the electrodes, R is the resistance obtained from impedance measurement and A is the cross sectional area of the gel.

### Data availability

Readers can access the data via contact to the authors.

## Electronic supplementary material

Supplementary Information

## References

[1] Kim TY (2011). High-Performance Supercapacitors Based on Poly(ionic Liquid)-Modified Graphene Electrodes..

[2] Arbizzani C (2008). Safe. High-Energy Supercapacitors Based on Solvent-Free Ionic Liquid Electrolytes. J. Power Sources.

[3] Ruiz V, Huynh T, Sivakkumar SR, Pandolfo AG (2012). Ionic Liquid–solvent Mixtures as Supercapacitor Electrolytes for Extreme Temperature Operation. RSC Adv..

[4] Balducci A, Bardi U, Caporali S, Mastragostino M, Soavi F (2004). Ionic Liquids for Hybrid Supercapacitors. Electrochem. commun..

[5] Kubisa P (2009). Ionic Liquids as Solvents for Polymerization Processes-Progress and Challenges. Prog. Polym. Sci..

[6] Kowsari, E. High-Performance Supercapacitors Based on Ionic Liquids and a Graphene Nanostructure. In *Ioinic Liquids-State of the Art;* Handy, S., Ed.; Intech, 2015.

[7] Taghavikish M, Subianto S, Dutta NK, Choudhury NR (2015). Facile Fabrication of Polymerizable Ionic Liquid Based-Gel Beads via Thiol–ene Chemistry. ACS Appl. Mater. Interfaces.

[8] Liew C-W, Ramesh S, Arof AK (2014). Good Prospect of Ionic Liquid Based-Poly(vinyl Alcohol) Polymer Electrolytes for Supercapacitors with Excellent Electrical, Electrochemical and Thermal Properties. Int. J. Hydrogen Energy.

[9] Yuan J, Mecerreyes D, Antonietti M (2013). Poly(ionic Liquid)s: An Update. Prog. Polym. Sci..

[10] Yuan J, Antonietti M (2011). Poly(ionic Liquid)s: Polymers Expanding Classical Property Profiles. Polymer.

[11] Thurecht KJ (2008). Free-Radical Polymerization in Ionic Liquids: The Case for a Protected Radical. Macromolecules.

[12] Lu J, Yan F, Texter J (2009). Advanced Applications of Ionic Liquids in Polymer Science. Prog. Polym. Sci..

[13] Huang C-W, Sun Y-ming (1997). Huang, Wei-fung, Curing Kinetics of the Synthesis of Poly (2-hydroxyethyl methacrylate) (PHEMA) with Ethylene Glycol Dimethacrylate (EGDMA) as a Crosslinking Agent. J. Polym. Sci. Polym. Chem..

[14] Demirelli, K., Cosë, M. & Kaya, E. A Detailed Study of Thermal Degradation of Poly (2-Hydroxyethyl Methacrylate). **72**, **7**5–80 (2001).

[15] Çekingen SK, Saltan F, Yildirim Y, Akat H (2012). A Novel HEMA-Derived Monomer and Copolymers Containing Side-Chain Thiophene Units: Synthesis, Characterization and Thermal Degradation Kinetics. Thermochim. Acta.

[16] Subianto S, Choudhury NR, Dutta NK (2008). Palladium-Catalyzed Phosphonation of SEBS. J. Polym. Sci. Part A Polym. Chem..

[17] Mistry MK, Subianto S, Choudhury NR, Dutta NK (2009). Interfacial Interactions in Aprotic Ionic Liquid Based Protonic Membrane and Its Correlation with High Temperature Conductivity and Thermal Properties. Langmuir.

[18] Pandey GP, Hashmi SA (2010). Kumar, Yogesh Performance Studies of Activated Charcoal Based Electrical Double Layer Capacitors with Ionic Liquid Gel Polymer Electrolytes. Energy Fuels.

[19] Visentin, A. F. & Panzer, M. J. Poly(Ethylene Glycol) Diacrylate-Supported Ionogels with Consistent Capacitive Behavior and Tunable Elastic Response. *ACS Appl*. *Mater*. *Interfaces* 4 2836–2839 (2 G. P. 012). 10.1021/am300372n 22583832

[20] Qin H, Panzer, Matthew J (2017). Chemically Cross-Linked Poly(2-hydroxyethyl methacrylate)-Supported Deep Eutectic Solvent Gel Electrolytes for Eco-Friendly Supercapacitors, Chem. Electro. Chem.

[21] Macfarlane DR (2007). Ionic Liquids in Electrochemical Devices and Processes: Managing Interfacial Electrochemistry. Acc. Chem. Res..

