# Supplementary material for: A Poly(ionic liquid) Gel Electrolyte for Efficient all Solid Electrochemical Double-Layer Capacitor
Source: Sci Rep. 2018 Jul 19;8:10918. doi: 10.1038/s41598-018-29028-y (PMC6053427; doi:10.1038/s41598-018-29028-y)
Supplement: Supplementary file 1 — Supplementary Information [file 41598_2018_29028_MOESM1_ESM.docx]

**SUPPORTING INFORMATION**

**NMR, FTIR, Phase angle plot of the electrochemical impedance of the PIL electrolyte; Galvanostatic charge-discharge curves, TGA curve of the IL.**

**A Poly(ionic liquid) Gel Electrolyte for Efficient all Solid Electrochemical Double-Layer Capacitor**

M. Taghavikish^a^, S.Subianto^a^, Y. Gu ^b^, X. Sun^b^, X. S. Zhao^b^, and N. Roy Choudhury^*a,c^

a University of South Australia, Mawson Lakes Campus, South Australia, Australia.

*University of Adelaide, Adelaide, South Australia, Australia

b University of Queensland, Brisbane, Australia

c School of Engineering, RMIT University, Melbourne, Victoria 3001, Australia

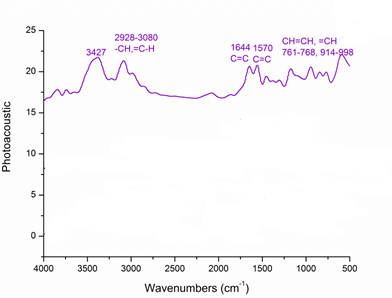


Figure S2.FTIR Spectrum of DVIMBr


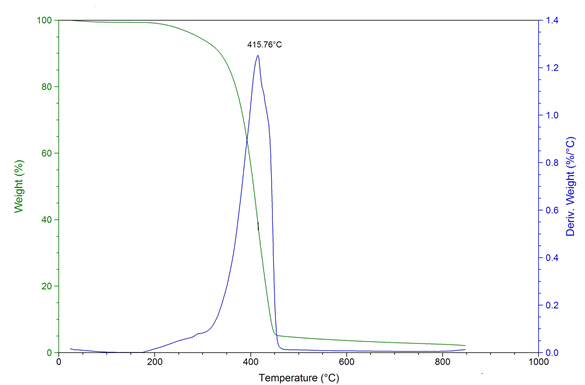


Figure S3. TGA of BMI-PF6

**Phase angle of the PIL gel electrolyte obtained from electrochemical impedance spectroscopy**

Figure S4 Phase evolution of samples containing 10% DVIMBr and 0,23,50,and 75% IL


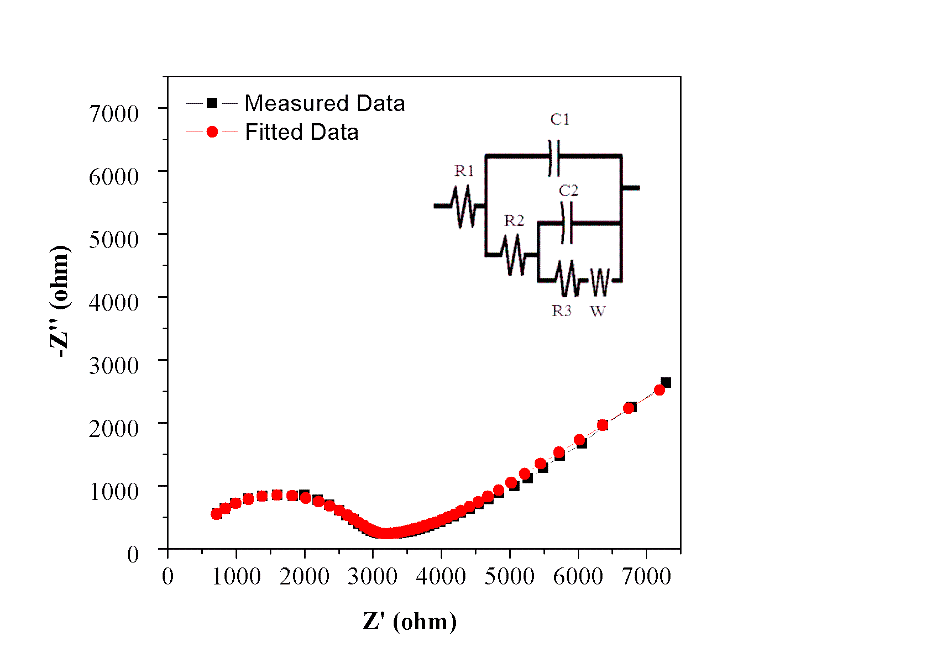


Figure S5 Nyquist plot (measured data and fitted data) of the sample containing 10% DVIMBr+ 75% IL (inset is the equivalent circuit diagram





Figure S6 Ragone plot of areal energy density versus areal power density for the device using as prepared polymerised ionic liquid as electrolyte.

**Charge Discharge curves for samples containing 10% DVIMBr**

Figure S7. GCD curves of sample with 0% IL.

Figure S8. GCD curves of sample with 23% IL

Figure S9. GCD curves of sample with 50% IL

Figure S10. GCD curves of sample with 75% IL


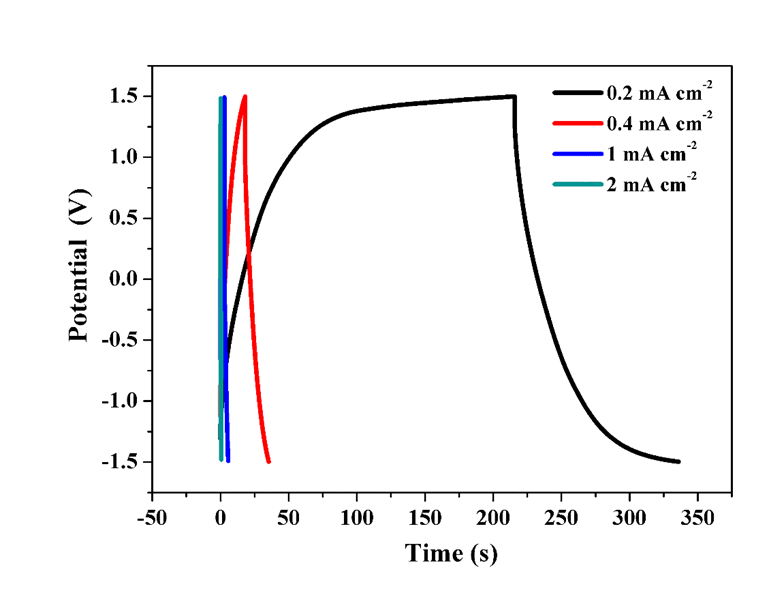


Figure S11. GCD curves of a supercapacitor cell fabricated with a gel containing 10% DVIMBr and 75% IL as electrolyte and commercial CNTs as both electrodes.
